# A simulation-based assessment of the efficiency of QTL mapping under environment and genotype x environment interaction effects

**DOI:** 10.1371/journal.pone.0295245

**Published:** 2023-11-30

**Authors:** Grace Sunshine David, José Marcelo Soriano Viana, Kaio Olimpio das Graças Dias

**Affiliations:** 1 Department of Crop Science, University of Calabar, Calabar, Cross River State, Nigeria; 2 Department of General Biology, Federal University of Viçosa, Viçosa, MG, Brazil; ICAR Indian Institute of Wheat and Barley Research, INDIA

## Abstract

The objective of this simulation-based study was to assess how genes, environments, and genotype x environment (GxE) interaction affect the quantitative trait loci (QTL) mapping efficiency. The simulation software performed 50 samplings of 300 recombinant inbred lines (RILs) from a F_2_, which were assessed in six environments. The RILs were genotyped for 977 single nucleotide polymorphisms (SNP) and phenotyped for grain yield. The average SNP density was 2 cM. We defined six QTLs and 190 minor genes. The trait heritability ranged from 30 to 80%. We fitted the single QTL model and the multiple QTL model on multiple phenotypes. The environment and complex GxE interaction effects led to a low correlation between the QTL heritability and power. The single- and across-environment analyses allowed all QTLs be declared, with an average power of 28 to 100%. In the across-environment analysis, five QTLs showed average power in the range 46 to 82%. Both models provided a good control of the false positive rate (6%, on average) and a precise localization of the QTLs (bias of 2 cM, on average). The QTL power in each environment has a high positive correlation with the range between QTL genotypes for the sum of the additive, environment, and GxE interaction effects (0.76 to 0.96). The uncertainty about the magnitude and sign of the environment and GxE interaction effects makes QTL mapping in multi-environment trials unpredictable. Unfortunately, this uncertainty has no solution because the geneticist has no control over the magnitude and sign of the environment and GxE interaction effects. However, the single- and across-environment analyses are efficient even under a low correlation between QTL heritability and power.

## Introduction

Because the negative implications in plant and animal breeding, decreasing the correlation between phenotype and genotype, as well as in the risk of developing many important human diseases, genotype x environment (GxE) interaction continues to be a significant research area over decades [1–3]. Several statistical approaches have been applied to understand the relative contributions of the genes, environments, and GxE interaction to phenotypes in two or more general or specific environments, to describe the phenotypic plasticity, and to minimize the influence of GxE interaction on selection. Using analysis of variance and two mice lines, and isolating the error variance, Dunn, O’Connell [4] observed that energy expenditure is highly dependent on genetic background (87 and 89%), whereas food intake and weight are highly dependent on diet (72 and 85%). Glucose tolerance and working memory showed a substantial interaction between genetic background and diet (22 and 44% and 38 and 39%, respectively). Describing adaptability (for plant breeders) or reaction norm (for animal breeders)–the expression pattern of a genotype or population along an environment gradient–allows cultivar/population management, but the approach is highly dependent on sample size and environmental range [2,3]. Taking into account the selection and the production environments, Mulder and Bijma [5] concluded that phenotyping relatives in both environments minimizes loss in genetic gain due to GxE interaction.

High genomic prediction accuracy has been achieved from multi-environment trials in several crops and animal populations by fitting marker x environment interaction, multiple traits, and environmental factors [6–8]. Because the statistical analysis is based on mixed linear models, the genome-wide association study (GWAS) allow efficient identification of QTLs and QTL x environment interaction [9,10]. Concerning quantitative trait loci (QTL) mapping, most investigations based on multi-environment trials along the last 10 years mapped QTLs in each environment and across environments, and tried to identify stable QTLs. Zhu, Weng [11] believe that stable QTLs are important to practical QTL-based breeding. According to Kushwah, Bhatia [12], stable QTLs for days to flowering in chickpea can be one of the major factors for providing heat tolerance. In general, a QTL was defined as stable when mapped in most environments. Wang, Sun [13] assumed additionally a phenotypic variance explained over 10%. Some authors also used the term partially stable.

The GxE interaction was commonly assessed by analysis of variance. In few studies the authors estimated the QTL x environment interaction effects or variance. Based on principal component analysis, Kato and Horibata [14] clearly observed that the response of the QTL additive effects to the environments was not randomly, but regulated by unknown internal mechanisms. They emphasize, however, that a better understanding of these mechanisms requires a proteomic analysis. Wang, Yang [15] observed that the ratios between QTL additive x environment and QTL additive variances corresponded to 0.1 to 22.5. In some studies the authors assessed QTL x environment interaction (QEI) by comparing the QTL additive effect or phenotypic variance explained across environments. For the QTLs with significant interaction with the well-watered and water stress environments, Li, Zhang [16] observed ratios between the additive QTL effects in the range 1 to 26. Chen, Zhao [17] emphasize that assessing QEI is a significant problem in QTL mapping. Their Bayesian model fit QTL effects in different environments, but no interaction effects. The interaction effects are represented by the variance of the QTL effects across environments. That is, the second moment parameter represents the degree of QEI. From the analyses of field and simulated datasets they concluded that the method is robust but the QEI may not be estimated accurately when the number of environments is small. The model proposed by Li, Wang [18] also fit only additive QTL effects in different environments. The QEI effect is estimated as the deviation between the QTL additive effect in an environment and the average additive effect across environments. The statistical approach is an extension of the composite interval method for multi-environment trials. From the analyses of simulated and field data, the authors recognized benefits to apply QEI analysis but cannot exclude the use of QTL mapping by each environment, and then summarize the mapping results across the environments.

In the simulation study of Chen, Zhao [17], the main focus was comparing covariance structures for the residual errors. In general, they observed some to many false positive main effects and that the estimated QTL effects and QTL x environment variance agreed well with the true values. But there is no information on QTL power of detection. Because the main objective of the simulation study of Li, Wang [18] was describing the additive QEI analysis, they simulated two simplified datasets, one including only two environments with equal heritability. In both simulation-based studies, no environment effect was specified. In the scenarios of two linked QTLs, Li, Wang [18] defined different additive effects for at least one QTL. Consequently, they added at least the interaction effect to the pure additive effects. Then, because there is limited information on the efficiency of QTL mapping in multi-environment trials, the objective of our simulation-based study was to assess how genes, environments, and GxE interaction affects the QTL power of detection, the false positive rate (FPR), and the mapping precision, assuming high SNP density, hundreds of minor genes, and very contrasting environmental conditions.

## Materials and methods

### Simulation

The simulated dataset was generated using the software *REALbreeding* (available on request). The program has been developed by the second author using Xojo (https://www.xojo.com/). There are versions for Windows, Linux, and MacOs. The current version allows the inclusion of digenic epistasis, genotype x environment interaction, and multiple traits, including pleiotropy. *REALbreeding* has been used in studies related to genomic selection, genome-wide association study, QTL mapping, linkage disequilibrium (LD), population structure, heterotic grouping/genetic diversity, quantitative genetics, and plant breeding. It can be also used for research in human genetics, animal genetics and breeding, population genetics, and evolution. The program simulates individual genotypes for genes and molecular markers and phenotypes in three stages, using inputs from the user. The first stage (genome simulation) is the specification of the number of chromosomes, molecular markers, and genes as well as marker type and density. The second stage (population simulation) is the specification of the population(s) and sample size or progeny number and size. A population is characterized by the average frequency for the genes (biallelic) and markers (first allele). In this stage, the user also defines the number of simulations (resamplings of the population). The final stage (trait simulation) is the specification of the minimum and maximum genotypic values for homozygotes, used to computing the parameters **m**, **a**, and **d** for each gene, the minimum and maximum phenotypic values (to avoid outliers), the direction and degree of dominance, and the broad sense heritability, used to compute the error variance.

It should be emphasized that the software does not sample the individual genetic values from a probability distribution. It computes the additive, dominance, and epistatic genetic values, the general and specific combining ability effects, or the genotypic values, depending on the population, from the **a** and **d** deviations, the gene frequencies–using a beta distribution–and the linkage disequilibrium (LD) values. Each initial population in LD is derived by crossing two populations in linkage equilibrium, followed by one generation of random crosses. The parametric LD in the gametic pool of this population is $\Delta_{ab}^{\left(0\right)}=(1-r_{ab})[(1-2r_{ab})/4](p_{a1}-p_{a2})(p_{b1}-p_{b2})$, where r is the recombination fraction, p is the allele frequency, and the indexes 1 and 2 refer to the parental populations. Detailed description of the simulation process can be found in Viana and Garcia [19].

In the first stage we defined 1,000 single nucleotide polymorphisms (SNPs), six QTLs (one on chromosome 1, two on chromosome 3, and three on chromosome 5), and 190 minor genes, distributed on 10 chromosomes of 200 cM (100 SNPs and 19 minor genes/chromosome). The minimum distance between linked QTLs was 20 cM. The software generated 977 SNPs, in the range 91 to 100 per chromosome. The average density was approximately 2 cM, as expected. In the second step we defined 300 RILs, six environments, and 50 simulations. To generate the RILs, the software crossed two inbred lines, assuming genes and SNPs in association, selfed the F_1_ plant, and derived 300 RILs using a single seed descent process (10 selfings). To simplify the dataset, avoiding, for example, defining two plots of 10 plants per environment (giving 50x300x6x2x10 = 1,800,000 plants), we requested a single RIL genotype in each environment (giving 50x300x6 = 90,000 entries), specifying, in the third stage, a heritability at the average plot level. In the third stage we defined additive-dominance model, 160 and 40 g/plant as the maximum and minimum genotypic values, 200 and 10 g/plant as the maximum and minimum phenotypic values, positive dominance (average degree of dominance between 0.0 and 1.2; 0.6 on average), and broad sense heritability in the range 30–80%. To investigate the significance of the GxE interaction, we generated a dataset with environment and GxE interaction effects and another only with environment effects.

The theoretical background includes the following assumptions regarding the environment and genotype x environment effects for each gene: (i) the sum of the environment effects for each genotype is zero; and (ii) the sum of the genotype x environment effects over the environments is zero, for each genotype. This implies that the population mean in a given environment is M + expectation(environment effect) + expectation(GxE interaction effect) and that the across environments population mean is M, since the sums of the environment and GxE interaction effects are zero. The environment and genotype x environment effects for each gene are sampled from a probability distribution, assuming $env∼N(0,\sigma_{env}^{2})$ and $gxe∼N(0,\sigma_{gxe}^{2})$. Thus, as an additional input from the user, the software requires the ratios $\sigma_{env}^{2}/\sigma_{g}^{2}$ and $\sigma_{gxe}^{2}/\sigma_{g}^{2}$, where $\sigma_{g}^{2}$ is the genotypic variance (single values, as 0.5 and 0.8, respectively, for example). We defined the ratios as one.

### Statistical analysis

The individual and across environment analyses were performed using the r/qtl package [20]. We did not generate a linkage map for each analysis. We used the genetic map provided by *REALbreeding*, with the true SNP positions in cM. We processed the single QTL model with the expectation-maximization method and the multiple QTL model on multiple phenotypes, using the phenotypic values in each environment as a different trait. The thresholds were obtained from 1,000 permutations.

### Efficiency of QTL mapping

For computing QTL detection power, FPR, and mapping precision (bias in the QTL positioning) from the r/qtl outputs, we used a *REALbreeding* tool (QTL summary). A declared QTL was assumed as a true QTL when the bias between its estimated and actual positions was lower than 20 cM.

## Results

**Fig 1:**
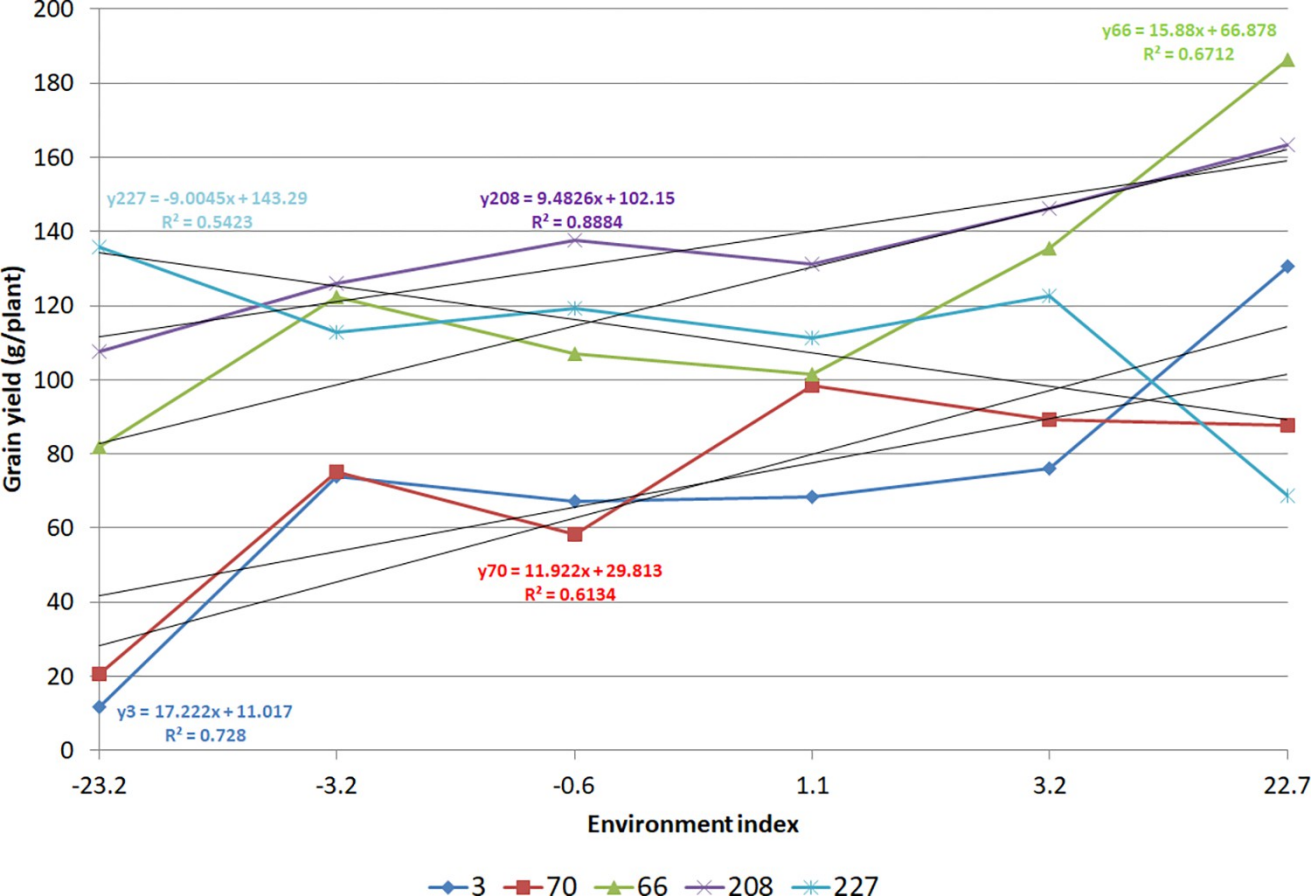
Reaction norm for five RILs showing complex GxE interaction.

The environment indexes, the environment and GxE interaction variances, and the trait heritabilities show sharp differences among the environments for the scenarios of GxE and no GxE interaction (Table 1). The reaction norms for few assessed RILs clearly show complex GxE interaction (Fig 1). Most of the correlations between these statistics were negative (in the range −0.89 to 0.76) for both scenarios. However, the correlations for the same statistics in the two scenarios were of high positive magnitude (0.84 to 1.0). The trait heritability ranged from low to high across the environments. The environment and the GxE interaction variances corresponded between 7 to 168% and 15 and 138% of the genotypic variance.

**Table 1:** Parents (P1 and P2), F1, and RILs average genotypic values (g/plant), environment index (Idx), genotypic $\left(\sigma_{g}^{2}),\;environment\;(\sigma_{env}^{2}),\;GxE\;interaction\;(\sigma_{gxe}^{2}\right)$, error $\left(\sigma^{2}),\;and\;phenotypic\;(\sigma_{p}^{2}\right)$ variances, and heritabilities (h^2^; %) in each environment, assuming GxE and no GxE interaction.

Scenario	P_1_	P_2_	F_1_	RIL	Env.	Idx	$\sigma_{g}^{2}$	$\sigma_{env}^{2}$	$\sigma_{gxe}^{2}$	$\sigma^{2}$	$\sigma_{p}^{2}$	h^2^
**GxE**	160.0	40.0	144.0	99.9	1	3.2	407.1	57.5	186.6	901.5	1355.1	30.0
					2	1.1	407.1	75.3	61.0	607.2	954.0	42.7
					3	−0.6	407.1	199.6	87.7	394.3	1032.0	39.5
					4	−3.2	407.1	124.1	63.7	270.0	479.2	84.9
					5	22.7	407.1	28.4	562.6	167.3	1453.5	28.0
					6	−23.2	407.1	683.7	104.0	129.4	1772.5	23.0
**No GxE**	160.0	40.0	144.0	99.9	1	2.8	407.1	57.5	-	891.0	1351.9	30.1
					2	1.1	407.1	75.3	-	599.9	1037.2	39.2
					3	9.1	407.1	199.6	-	394.6	831.0	49.0
					4	−1.7	407.1	124.1	-	271.0	482.4	84.0
					5	11.2	407.1	28.4	-	171.0	700.5	58.1
					6	−22.5	407.1	683.7	-	98.8	1482.3	27.5
**GxE**	160.0	40.0	144.0	99.9	1	−21.1	407.1	171.2	39.8	948.6	1339.3	30.3
					2	18.8	407.1	23.1	938.8	609.8	2158.4	18.8
					3	−5.6	407.1	401.1	251.5	406.5	1535.2	26.5
					4	21.6	407.1	71.8	71.0	271.0	561.3	72.4
					5	−14.4	407.1	28.7	65.7	174.2	630.3	64.5
					6	0.73	407.1	116.8	123.2	101.6	279.2	80.0

**Fig 2:**
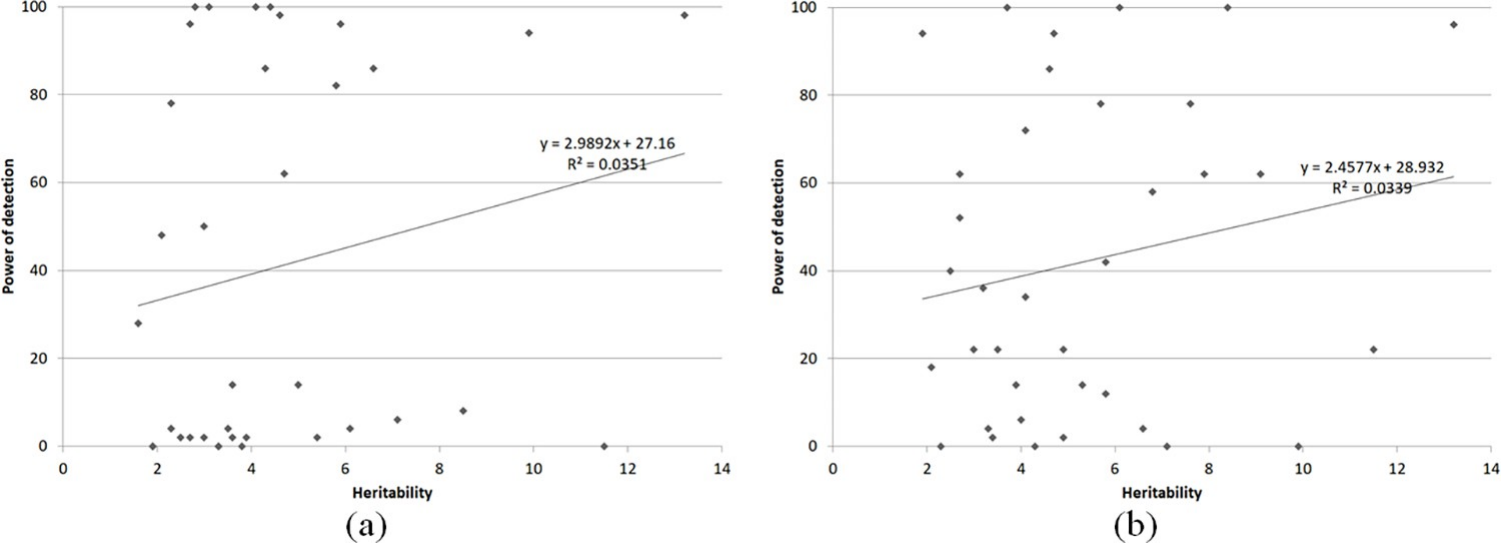
Heritabilities and power of detection for six QTLs in six environments, assuming GxE (a) and no GxE (b) interaction and fitting the single QTL model.

A consequence of the environment and the GxE interaction effects was a low correlation between the QTL heritability and power of detection (approximately 0.2) for both scenarios (Table 2 and Fig 2), from the single QTL model analyses. Thus, even assuming no GxE interaction, the environment effect had a significant influence on the QTL mapping, decreasing the correlation between the QTL heritability and power of detection. Including GxE interaction, the QTL mapping efficiency decreased even more: in 75% of the environments the correlation between the QTL heritability and power of detection decreased 37 to 80%, the number of QTLs detected in all environments decreased 30%, and the QTL power of detection decreased for 50% of the QTLs. Concerning the FPR and the mapping precision, the single QTL model provided a good control of the type I error (2 to 11%; approximately 6%, on average) and a precise localization of the QTLs (bias from 1.1 to 2.9 cM; approximately 2 cM, on average), for both scenarios. Interestingly to emphasize, the correlations between the FPR and bias in the positioning the QTLs with environment and GxE variances were negative, regardless of the GxE interaction. The analysis across environments showed no significant differences between the scenarios of GxE and no GxE interaction, showing no correlation between the QTL heritability and power of detection (0.01), effective control of the FPR (approximately 4%), and very precise mapping (approximately 2 cM).

**Table 2:** QTL heritability (h^2^; %) and average QTL power of detection (%), FPR (%), and bias (cM) in the positioning the true QTLs, in each environment and across the environments, assuming GxE and no GxE interaction and fitting the single QTL and the multi-trait models (values after /).

Env.	QTL	GxE				No GxE			
		h^2^	Power	FPR	Bias	h^2^	Power	FPR	Bias
1	1	2.1	48/42	6.3/4.0	2.9/2.7	2.1	18/20	5.4/6.5	2.7/2.6
	2	3.5	4/2			3.5	22/20		
	3	3.0	2/0			3.0	22/18		
	4	4.7	62/62			4.7	94/94		
	5	4.1	100/96			4.1	72/68		
	6	2.5	2/6			2.5	40/36		
2	1	3.0	50/46	8.2/5.5	2.9/3.0	2.7	62/62	7.7/5.5	2.5/2.4
	2	5.0	14/12			4.6	86/86		
	3	4.3	86/84			3.9	14/14		
	4	6.6	86/80			6.1	100/100		
	5	5.8	82/78			5.3	14/12		
	6	3.6	14/10			3.3	4/4		
3	1	2.7	2/2	2.0/3.0	1.2/1.6	3.4	2/2	3.2/1.0	2.1/2.1
	2	4.6	98/96			5.7	78/78		
	3	3.9	2/0			4.9	22/22		
	4	6.1	4/4			7.6	78/74		
	5	5.4	2/2			6.6	4/4		
	6	3.3	0/0			4.1	34/28		
4	1	5.9	96/92	8.5/8.3	2.0/1.7	5.8	12/10	11.1/6.5	2.0/2.6
	2	9.9	94/94			9.9	0/0		
	3	8.5	8/4			8.4	100/100		
	4	13.2	98/94			13.2	96/92		
	5	11.5	0/0			11.5	22/20		
	6	7.1	6/4			7.1	0/0		
5	1	1.9	0/0	4.5/2.7	1.1/1.3	4.0	6/4	4.7/4.3	1.9/1.9
	2	3.3	0/0			6.8	58/60		
	3	2.8	100/96			5.8	42/40		
	4	4.4	100/96			9.1	62/64		
	5	3.8	0/0			7.9	62/58		
	6	2.3	78/72			4.9	2/2		
6	1	1.6	28/22	2.3/2.7	1.8/1.8	1.9	94/94	3.2/2.0	2.1/1.9
	2	2.7	96/92			3.2	36/34		
	3	2.3	4/4			2.7	52/52		
	4	3.6	2/2			4.3	0/0		
	5	3.1	100/96			3.7	100/100		
	6	1.9	0/0			2.3	0/0		
Across	1	2.1	82	4.5	2.1	2.6	86	4.4	2.2
	2	3.5	48			4.3	44		
	3	3.0	52			3.7	56		
	4	4.7	46			5.8	52		
	5	4.1	72			5.0	74		
	6	2.5	14			3.1	16		

The multiple QTL model on multiple phenotypes provided essentially the same results from the single QTL model (Table 2). The correlations between the values for the QTL power of detection, FPR, and bias in the QTL positioning were 1.0, 0.8, and 0.9, assuming GxE interaction, and 1.0, 0.8, and 0.6, assuming no GxE interaction, respectively. The single QTL model provided a slight higher power; on average, increases of 9 and 3% for the scenarios of GxE and no GxE, respectively. In consequence, this model provided a higher FPR; on average, increases of 7 and 27% for the scenarios of GxE and no GxE, respectively. The mapping precision showed unimportant differences; on average, −5.5 and −2.5% for the two scenarios, in favor of the single QTL model.

**Fig 3:**
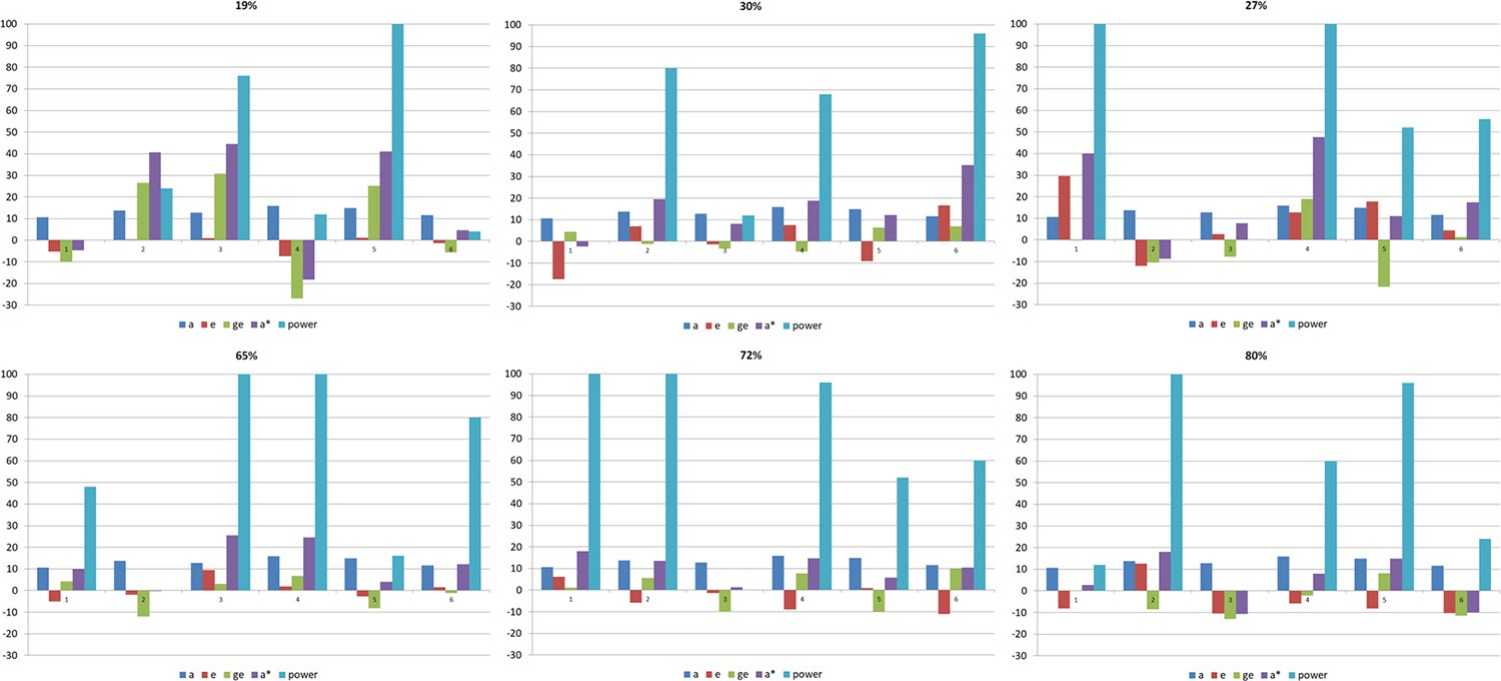
Ranges between genotypes for the additive (a), environment (e), GxE interaction (ge) effects, and sum of the effects (a*), for six QTLs in six environments, characterized by the trait heritability.

For a detailed assessment of the relative influence of the QTL additive, environment, and GxE interaction effects on the power of detection, we analyzed the true values provided by *REALbreeding*. Because we accidentally lost the output files with the environment and GxE interaction effects for the QTLs, we simulated a new comparable dataset, but with new random environment and GxE interaction effects (same gene effects) (Table 1). We observed that the range for the two homozygous genotypes, regarding the additive (a), the environment (e), the GxE interaction (ge) effects, and the sum of these effects (a*) correlates with the QTL power of detection, allowing assess their relative importance (Fig 3). The QTL power of detection in each environment has a high positive correlation with the range for the sum of the additive, environment, and GxE interaction effects (between 0.76 and 0.96). The correlation between the range for the additive effect and power of detection varied from −0.04 to 0.69. Concerning the correlations between the ranges for the environment and the GxE interaction effects and power of detection, 5/6 of the values varied from 0.66 to 0.93 and 0.48 to 0.86, respectively. Regressing the QTL power of detection on each environment on the ranges for the additive, environment, and GxE interaction effects confirmed that the main factors affecting the QTL power of detection were environment and GxE interaction. Only in the environment with trait heritability of 72% the additive effect showed a significant effect on power.

## Discussion

To minimize the GxE interaction negative influence, increasing genetic gains and prediction accuracy or allowing efficient cultivar/population management, breeders developed methods to separate the environment and interaction effects from the genetic effects, to describe the adaptability or reaction norm, to identify genotypes close to a ideotype, and to cluster the main sites of assessment [3,21–23]. The many adaptability/reaction norm studies proves that genotypes does not respond to environment in a randomly way. Instead, as demonstrated by Kato and Horibata [12], the response is predictable, although the genetic, biochemical, and physiological mechanisms requires further investigation based on transcriptomic, proteomic, and metabolomic analyses for specific major genes determining quantitative traits [24–26].

Unfortunately, environment and especially GxE interaction are abstract concepts, of difficult or impossible control when genotypes are assessed in multi-environment trials (MET). A QTL mapping can be performed under two sowing densities or at two contrasting levels of water availability but this does not mean that the scientist effectively controlled the environment. The QTL mapping in MET has shown a significant influence of environment and GxE interaction on phenotypes. In the study of Kato and Horibata [12], using rice near isogenic lines distinct for four single- and five double-QTLs, the analysis of variance for the grain length showed higher variability for the genetic background and lower variability for the interaction effects. Higher genotypic variance, intermediate environment variance, and lower GxE interaction variance was also observed in the study of Wang, Yang [15], for the ratio of multi-seed pods per plant in peanut RILs. Based on a pooled analysis of two sites, Kushwah, Bhatia [12] observed in chickpea that the yield per plant genotypic variance was 3 and 11 times higher than the GxE interaction variance, in two sowing dates. In many QTL mapping studies in MET there is no information on the relative significance of the environment effect because the authors assumed environment as a fixed effect. However, the variability for the phenotypic variance explained (PVE) regarding the same QTL or for the QTL additive effects in distinct environments also shows that the environment was an important factor affecting the QTL mapping efficacy. A QTL for thousand kernel weight mapped in two environments by Haugrud, Zhang [27] showed a PVE ratio of 1.8. For one of the QTLs mapped by Li, Zhang [16] under well-watered and water stress conditions, the ratio between their estimated additive effects was 23. Sixty percent of the QTLs showed different signs, a result clearly explained by our investigation.

The difference in sign of the estimated QTL additive effects is because the analysis based on RILs or doubled-haploid lines provides estimation of the additive effect plus the environment and the QTL x environment interaction effects, not the pure additive effect. In the MET analysis using F_2:3_ or selfed backcross progeny, both additive and dominance QTL effects are affected by environment and QTL x environment interaction. Thus, differences of sign are expected even for epistatic effects in an epistatic QTL mapping in MET. Based on our results and also on the results from many field QTL mapping over environments, we can state that the QTL mapping in MET is not a challenger. In these studies, there are several declared QTLs for each trait, most of them unstable [28,29]. However, because the significance of the environment and QTL x environment interaction effects, we can characterize the QTL mapping over environments as unpredictable, assuming a low correlation between environment index and trait heritability. Note that it is possible that a QTL with a significant additive effect (high heritability or PVE) do not be declared in one of the environments if the range between genotypes for the sum of additive, environment, and QTL x environment interaction effects is close to zero. However, because the RILs, or other mapping population, were assessed in at least two contrasting environments, the same QTL will be declared if the range for the sum of additive, environment, and QTL x environment interaction effects is higher than the additive effect or if the range for the sum of environment and QTL x environment interaction effects is close to zero. Thus, even in contrasting environments with significant environment and GxE interaction effects we showed that all QTLs were mapped in at least one environment and most were mapped in the across environment analysis.

In regard to the efficiency of QTL mapping in MET, it is important to emphasize that, even assuming a low correlation between the trait heritability, in the range 30 to 85%, and the environment index, in the range −23 to 23 g/plant, the environment and GxE interaction effects in the single environment analyses allowed all QTLs be declared, with an average power of detection in the range 28 to 100%. In the across environment analysis, five out six QTLs showed an average power of detection in the range 46 to 82%. This is an impressive positive result, although unpredictable because the breeder does not know how to predict if the environment and QTL x environment will increase the range between genotypes for the sum of pure additive and dominance, environment, and GxE interaction effects. Other significant result is that the environment and genotype x environment effects does not lead to a higher FPR, due to minor genes [30]. The FPR in the single- and across-environment analyses are within the expectation, based on other simulation-based studies in a single environment [31,32]. With respect to the mapping precision, no negative result was observed in this study, irrespective of the model and the magnitude of the environment and GxE interaction effects. All mapped QTLs showed a bias in the true position in the range of approximately 1 to 3 cM. Assuming five independent QTLs, no minor genes, and defining two environments with heritability of 50%, Li, Wang [18] observed very high power of detection for all QTLs, ranging from 80.7 to 98.3%, FPR of 14.1%, and a bias in the QTL position of only 0.2 cM. For the scenarios including two linked QTLs, the QEI mapping efficiency was proportional to the trait heritability. The authors observed power of detection higher than 80% assuming heritability of 50 and 80%, but in the range 10 to 65% assuming heritability of 10%. The FPR was 10% for heritability of 80% but 30 to 50% for heritability of 10%.

Concerning the statistical approach for QTL mapping in MET, Li, Wang [18] emphasize that the QEI mapping allows assessing QTL stability and QEI effect. However, no significant advantage comparative to single- and across-environment analyses was observed. Our results showed no significant difference between the single QTL and the multi-trait models. Concluding, the uncertainty about the magnitude and sign of the environment and GxE interaction effects makes the QTL mapping in MET unpredictable, since these effects decreases the correlation between QTL heritability and power of detection. Unfortunately, this uncertainty has no solution because the geneticist has no control over the magnitude and sign of the environment and GxE interaction effects. However, the single- and across-environment analyses based on the single- or multiple QTL model, under very different environment gradient, provided the identification of all QTLs, with effective control of the type I error and precise mapping. The search for a stable QTL, one that is mapped regardless of the environment seems no sense to us because, as demonstrated, a QTL only will be mapped in all or in most of the environments if the combined effects of QTL, environment, and QTL x environment interaction increases the range between genotypes. However, this is beyond the breeder’s control.
